# Дефицит витамина D у пациентов с избыточной массой тела: современные стратегии и практические аспекты

**DOI:** 10.14341/probl13557

**Published:** 2025-01-27

**Authors:** Т. Л. Каронова, В. В. Салухов, Ф. Х. Дзгоева, Е. А. Пигарова, Г. Р. Галстян, С. В. Булгакова, Г. Р. Вагапова, Н. И. Волкова, Т. П. Киселева, Т. Н. Маркова, О. В. Ремизов, Л. А. Скакун, В. Л. Тюльганова, В. В. Являнская

**Affiliations:** Национальный медицинский исследовательский центр им. В.А. Алмазова; Первый Санкт-Петербургский государственный медицинский университет им. И.П. Павлова; Военно-медицинская академия имени С.М. Кирова; Национальный медицинский исследовательский центр эндокринологии; Национальный медицинский исследовательский центр эндокринологии; Национальный медицинский исследовательский центр эндокринологии; Самарский государственный медицинский университет; Казанский государственный медицинский университет; Ростовский государственный медицинский университет; Уральский государственный медицинский университет; Эндокринологический диспансер Департамента здравоохранения г. Москвы; Российский университет медицины; Северо-Осетинская государственная медицинская академия; Городская больница №11; Южно-Уральский государственный медицинский университет; Челябинская областная клиническая больница; Южно-Уральский государственный медицинский университет; Кубанский государственный медицинский университет; Краевая клиническая больница №2

**Keywords:** витамин D, недостаточность, дефицит, избыточная масса тела, ожирение, сахарный диабет 2 типа, колекальциферол

## Abstract

В г. Владикавказе 27 сентября 2024 г. состоялось рабочее совещание в дискуссионном формате по проблеме дефицита витамина D у пациентов с избыточной массой тела и ожирением.

Цель заседания — оценить взаимосвязь дефицита витамина D с избыточной массой тела и коморбидными нарушениями, рассмотреть современные стратегии и практические аспекты ведения таких пациентов в практике эндокринолога.

Резолюцию рабочего совещания разработали его участники — ведущие специалисты-эндокринологи.

## Abstract (en)

On September 27, 2024, a discussion-based working meeting on the issue of vitamin D deficiency in patients with overweight and obesity was held in Vladikavkaz.The meeting aimed to evaluate the relationship between vitamin D deficiency, overweight, and associated comorbidities, as well as to explore modern strategies and practical approaches for managing such patients in endocrinology practice. The resolution of the meeting was developed by its participants, comprising leading endocrinologists.

Известно, что витамин D относится к важным микронутриентам, регулирующим не только костно-минеральный обмен, но и поддерживающим функционирование многих систем организма [1–4]. По мере изучения и подтверждения многогранности функций витамина D все более актуальным становится разработка стратегий, направленных на уменьшение встречаемости дефицита/недостаточности витамина D в популяции. Сегодня выделяют ряд факторов, включая ограниченное воздействие солнечного света, недостаточное питание, заболевания ЖКТ, социальные, а также экономические и национальные факторы, которые ассоциированы с низким уровнем обеспеченности данным нутриентом [5–8].

В последнее десятилетие внимание специалистов сфокусировано на вопросах потенциального влияния витамина D на развитие и течение ряда эндокринных, включая ожирение и сахарный диабет (СД), а также аутоиммунных и онкологических заболеваний [9–13].

Ожирение представляет собой многофакторное хроническое рецидивирующее заболевание с положительным энергетическим балансом и избыточным накоплением жира, которое способствует снижению качества и продолжительности жизни [14–16]. За последние 50 лет показатели распространенности ожирения во всем мире неуклонно растут, принимая характер пандемии, что негативно сказывается на общественном здравоохранении и экономике. Так, результаты ранее проведенных исследований показали, что более 650 миллионов взрослых (примерно 13% взрослого населения мира) страдают ожирением, а среди детей и подростков в возрасте от 5 до 19 лет его распространенность достигает более 340 миллионов [17][18].

## СВЯЗЬ ДЕФИЦИТА ВИТАМИНА D И ОЖИРЕНИЯ

Наблюдающийся параллельный рост распространенности ожирения и дефицита витамина D в популяции может быть объяснен общим этиологическим фактором, а именно современным образом жизни, который характеризуется низкой физической активностью, ограниченным временем пребывания на солнце с дополнительным использованием солнцезащитных кремов, а также пищевыми привычками, включая потребление высококалорийных продуктов, не содержащих витамин D или нарушающих его всасывание. Наличие у большинства больных ожирением неалкогольной жировой болезни печени (НАЖБП) также негативно сказывается на обеспеченности витамином D за счет уменьшения активности гидроксилаз печени, принимающих участие в образовании транспортной формы витамина D — 25-гидроксивитамина D (25(OH)D) или кальцидиола. Дополнительно при избыточном количестве жировой ткани наблюдается уменьшение биодоступности витамина D вследствие его секвестрации в жировых депо организма как высоколипофильного вещества [19]. Несмотря на это, следует учитывать тот факт, что уменьшение объема жирового депо при снижении массы тела не приводит к увеличению концентрации 25(ОН)D в сыворотке крови в связи с тем, что витамин D разрушается до неактивных форм и не поступает в кровоток [20]. Растущая популярность метаболической (бариатрической) хирургии, часто сопровождающейся развитием синдрома мальабсорбции, может усугублять недостаточность витамина D [21–23], особенно если статус витамина D остался без должного внимания до или после оперативного лечения.

Связь дефицита витамина D с ожирением и ассоциированными с ним заболеваниями (СД, сердечно-сосудистые заболевания (ССЗ), остеоартрит и др.) обсуждается в ряде метаанализов [24–28]. Установлено, что в печени, жировой ткани и мышцах витамин D способен проявлять противовоспалительное действие. Данный факт подтверждается уменьшением метилирования генов адипокинов, связанных с воспалением, а также инфильтрации макрофагами в условиях дефицита витамина D. В то же время нормализация уровня витамина D, наоборот, ассоциирована со снижением воспалительной реакции в висцеральной жировой клетчатке [29][30]. В совокупности эти данные подчеркивают важное значение дефицита витамина D в активации процессов воспаления в висцеральном жире, а также роль этого нутриента в качестве важного фактора регуляции в патогенезе ожирения [31].

Следует отметить и важность генетически детерминированного состояния рецепторов витамина D (VDR), которые принимают участие в пролиферации и гипертрофии адипоцитов [32]. Исследовательские работы на жировой ткани животных и человека in vitro показали, что витамин D оказывает разнонаправленное (стимулирующее или ингибирующее) воздействие на адипогенез посредством влияния на факторы транскрипции и модуляции экспрессии генов [33]. Так, результаты исследований показали влияние витамина D посредством VDR-зависимого подавления экспрессии PPARγ (рецептор, активируемого пролифератором пероксисом, гамма) [34]. Таким образом, накопленные на сегодняшний день данные свидетельствуют в пользу важной патофизиологической роли витамина D в дифференцировке жировой ткани.

## СВЯЗЬ ДЕФИЦИТА ВИТАМИНА D И ОЖИРЕНИЯ С НАРУШЕНИЕМ УГЛЕВОДНОГО ОБМЕНА

Известно, что дефицит витамина D у пациентов с ожирением, особенно в сочетании с сахарным диабетом типа 2 (СД2), является фактором, повышающим вероятность развития и прогрессирования осложнений данных заболеваний [35][36]. При этом весьма ограничено количество доклинических и клинических исследований, проведенных с целью изучения взаимосвязи между уровнем обеспеченности витамином D и СД2. Перекрестные исследования при этом подтверждают положительную связь между уровнем 25(OH)D в сыворотке крови и показателем чувствительности тканей к инсулину и индексом инсулинорезистентности при проведении гиперинсулинемического эугликемического клэмпа [37]. Авторы объясняют такую связь увеличением экспрессии VDR в мышцах, печени и жировой ткани с последующей активацией транскрипции генов, увеличивающих содержание (в экспериментальных моделях в 2,4 раза) субстрата рецепторов инсулина (IRS) в органах-мишенях и улучшением клеточного транспорта глюкозы [26]. Дополнительно, активная форма витамина D — кальцитриол (1,25(ОН)2D), повышая экспрессию кальбиндина, может снижать цитокин-индуцированный апоптоз β-клеток, а также уменьшать накопление продуктов гликирования, что в свою очередь ассоциировано с уменьшением резистентности к инсулину и снижением риска развития осложнений СД2 [14].

Анализ литературы свидетельствует о наличии данных в пользу улучшения чувствительности к инсулину за счет повышения 1,25(ОН)2D — опосредованной экспрессии рецепторов инсулина и увеличения активации PPAR-δ (рецептора, активируемого пролифератором пероксисом, дельта). Примечательно, что PPAR-δ принимает участие в регуляции метаболизма жирных кислот в жировой ткани и скелетных мышцах. Также установлена роль витамина D в секреции инсулина (поскольку 1,25(ОН)2D выступает в роли химического посредника, регулирующего поток кальция в β-клетки поджелудочной железы). Таким образом, дефицит витамина D может приводить к снижению секреции инсулина [37][38].

Вышеизложенные сведения о важной роли витамина D в регуляции секреции инсулина и чувствительности к нему в тканях и органах-мишенях, а также возможные механизмы участия в модуляции процессов воспаления и метаболизме липидов в случае дефицита подтверждают участие витамина D в развитии нарушений углеводного обмена, включая СД2.

## СВЯЗЬ ДЕФИЦИТА ВИТАМИНА D С НАРУШЕНИЕМ РЕПРОДУКТИВНОГО ЗДОРОВЬЯ

Как известно, ожирение ассоциировано с развитием нарушений репродуктивного здоровья у женщин, которые включают в себя нарушения менструального цикла, синдром поликистозных яичников, эндометриоз, более ранее наступление менопаузы, бесплодие, развитие гиперпластических процессов эндометрия и препятствие к использованию вспомогательных репродуктивных технологий, а также с развитие гипогонадизма у мужчин. Целый ряд исследований подтвердил тот факт, что на фертильность женщин и мужчин может оказывать влияние дефицит витамина D, поскольку присутствие рецепторов витамина D и ферментов, участвующих в его метаболизме, обнаружено в органах репродуктивной системы и сперматозоидах человека. Полагают, что влияние витамина D на органы-мишени возможно как напрямую — посредством связывания со своими рецепторами, так и опосредованно, — через стимуляцию синтеза стероидных гормонов (прогестерона, эстрогенов и тестостерона), необходимых для полноценного созревания фолликулов и эндометрия [39–42]. Исследование О.А. Громовой и соавт. показало регуляцию эндометриальной экспрессии гена HOXA10 витамином D, который необходим для процесса имплантации, а также участвует во взаимодействии эмбриона и эндометрия посредством различных молекулярных и цитокиновых механизмов. Все это в итоге способствует успешной имплантации эмбриона. Результаты указанного исследования продемонстрировали, что достаточный уровень 25(OH)D в сыворотке крови (более 30 нг/мл) улучшает результаты ВРТ по количеству достигнутых клинических беременностей [43]. В последнее время появляются данные, свидетельствующие и о положительном влиянии терапии препаратами витамина D на овуляцию и частоту наступления беременности у женщин с синдромом поликистозных яичников [44][45]. Между тем вопрос, оказывает ли прием витамина D положительное влияние на другие результаты ЭКО, включая частоту оплодотворения и продолжающуюся беременность, требует дальнейшего изучения. Кроме того, крайне важно определить, какие формы и дозы витамина D могут быть оптимальными во время цикла ЭКО, поскольку в настоящее время такие исследования отсутствуют [46].

Современные исследования демонстрируют частое сочетание дефицита витамина D с мужским бесплодием, низким уровнем тестостерона, а низкие значения 25(OH)D крови — с низким качеством спермы. Однако, несмотря на полученные результаты, на сегодняшний день все еще нет убедительных доказательств положительного влияния приема препаратов витамина D на показатели спермы и гормональные изменения, что подтверждает необходимость проведения дополнительных клинических исследований в этой области [47].

## СВЯЗЬ ДЕФИЦИТА ВИТАМИНА D И ОЖИРЕНИЯ С ИММУНОЛОГИЧЕСКИМИ ПАРАМЕТРАМИ

Исследования последних лет продемонстрировали, что наличие ожирения ассоциировано с повышенным риском развития некоторых острых респираторных вирусных инфекций (ОРВИ), в том числе гриппа, респираторно-синцитиальной вирусной инфекции, а также COVID-19. Так, было показано, что наличие избыточной массы тела отрицательно влияет на гуморальное звено иммунитета, а также ассоциировано с развитием критических осложнений вирусных инфекций [48][49]. Накоплены данные, свидетельствующие об участии витамина D в активации врожденного и приобретенного иммунного ответа. Что обусловлено наличием в иммунных клетках экспрессии гена CYP27B1, кодирующего фермент 1-альфа-гидроксилазу [50]. Одна из функций витамина D связана с локальным увеличением концентрации кальцитриола при ОРВИ и других инфекционных заболеваниях за счет индукции синтеза антимикробных пептидов, таких как дефензины и кателицидин LL-37 [51][52]. Для витамина D дополнительно описано подавление пролиферации и дифференцировки Т-хелперов (Th) со снижением уровня Th17, а также изменением баланса между Th2 и Th1, что способствует снижению экспрессии генов провоспалительных цитокинов и синтезу противовоспалительных цитокинов [53].

Популяционные исследования демонстрируют, что наиболее низкий уровень 25(OH)D в сыворотке крови регистрируется в зимние и весенние месяцы [54], что совпадает с повышением заболеваемости ОРВИ. При этом результаты ранее проведенных исследований демонстрируют снижение заболеваемости и тяжести течения ОРВИ у лиц с нормальным уровнем обеспеченности витамином D [55]. Результаты исследования, проведенного в Великобритании, показали снижение заболеваемости ОРВИ на 7% при повышении уровня 25(OH)D в сыворотке крови на каждые 10 нмоль/л [56]. Исследования, проведенные во время пандемии, также продемонстрировали связь между дефицитом витамина D и более тяжелым течением COVID-19 [57], а также положительный эффект терапии колекальциферолом на клиническое течение заболевания и уровень воспалительных маркеров [58][59]. Следует отметить, что положительные эффекты от данной терапии наблюдались у больных независимо от их массы тела.

Таким образом, принимая во внимание известные иммуномодулирующие эффекты витамина D, накопленные данные о положительном влиянии терапии колекальциферолом, можно рассматривать дефицит витамина D у больных ожирением как дополнительный модифицируемый фактор, оказывающий влияние на течение ОРВИ, в том числе COVID-19.

## ВОЗМОЖНОСТИ ИСПОЛЬЗОВАНИЯ ПРЕПАРАТОВ ВИТАМИНА D У БОЛЬНЫХ ОЖИРЕНИЕМ И АССОЦИИРОВАННЫМИ С НИМ ЗАБОЛЕВАНИЯМИ

Согласно клиническим рекомендациям Российской ассоциации эндокринологов (2021 г.), диагностика дефицита витамина D проводится с помощью определения в крови уровня 25(ОН)D, суммарного для двух форм витамина — D3 и D2 [60]. Для профилактики дефицита витамина D у взрослых рекомендуется его прием в дозе 800–1000 МЕ в сутки. В то же время при выявлении дефицита витамина D (25(ОН)D<20 нг/мл) лечение взрослых пациентов рекомендуется начинать с суммарной насыщающей дозы колекальциферола 400 000 МЕ, часто распределенной на 8 недель, а для коррекции недостаточности (уровень 25(ОН)D в сыворотке крови в диапазоне 20–30 нг/мл) использовать половину этой дозы (200 000 МЕ), как правило распределенной на 4 недели, с дальнейшим переходом на поддерживающие дозы 1000–2000 МЕ в сутки для сохранения 25(ОН)D в целевом диапазоне.

Выбор схемы лечения (ежедневный, еженедельный, ежемесячный прием) определяется индивидуально с учетом предпочтений пациента, а также максимальной ожидаемой приверженности к лечению. Пациентам с ожирением, НАЖБП, синдромами мальабсорбции, а также принимающим препараты, нарушающие метаболизм желчных кислот и витамина D, может потребоваться прием в 2–3 раза более высоких доз колекальциферола с последующим переходом на поддерживающую дозу (не менее 3000–6000 МЕ в сутки) для достижения целевого уровня 25(ОН)D [60].

Целевой диапазон показателя 25(ОН)D крови на фоне терапии до сих пор остается предметом дискуссий. Так, для реализации витамином D его иммуномодулирующих свойств нередко обсуждается концентрация 25(OH)D в сыворотке крови в диапазоне 30–50 нг/мл [61]. В двойном слепом плацебо контролируемом рандомизированном клиническом исследовании у пациентов с ожирением и дефицитом витамина D лечение колекальциферолом в дозе 50 000 МЕ в неделю в течение трех месяцев оказало положительное влияние на снижение массы тела и уменьшение жировой массы [62]. Параллельно увеличению концентрации 25(ОН)D в сыворотке крови отмечалось снижение ПТГ и провоспалительных маркеров крови. Таким образом, полученные результаты позволяют подтвердить возможность эффективного управления массой тела на фоне повышения уровня 25(ОН)D крови при использовании колекальциферола в дозе 50 000 МЕ в неделю [62].

В настоящее время к лекарственным препаратам колекальциферола, используемым для профилактики и лечения недостаточности/дефицита витамина D, относится препарат Солигамма®, который выпускается в виде таблеток малого размера, покрытых пленочной оболочкой, в дозировке 10 000 МЕ. При дефиците витамина D взрослым рекомендуется прием пяти таблеток Солигамма® 1 раз в неделю в течение 8 недель, что соответствует терапевтической дозе 400 000 МЕ. Коррекция недостаточности витамина D может проводиться при помощи приема пяти таблеток 1 раз в неделю в течение 4 недель, что соответствует суммарной дозе 200 000 МЕ [63]. В случае лечения пациентов с синдромом мальабсорбции, ожирением или лиц, принимающих препараты, нарушающие всасывание или метаболизм витамина D, доза витамина D должна быть увеличена в 2–3 раза или увеличение дозы может происходить с помощью повышения длительности приема препарата. При этом второй вариант для ряда пациентов может носить приоритетный характер. Необходимо отметить тот факт, что перед назначением терапевтических (насыщающих) доз витамина D требуется исключить наличие у больного первичного гиперпаратиреоза или гранулематозных заболеваний, при которых терапия колекальциферолом может привести к гиперкальциемии. Для поддержания достигнутых целевых уровней 25(ОН)D крови целесообразно продолжать прием Солигамма® по одной таблетке в дозе 10 000 МЕ один раз в неделю [60]. Такой вариант терапии показал одинаковую эффективность по сравнению с приемом масляных капель витамина D в отношении повышения концентрации 25(OH)D [64]. Назначение профилактических поддерживающих доз обычно не требует контроля показателей фосфорно-кальциевого обмена (ПТГ, Са общий, альбумин, креатинин, фосфор), а контроль уровня 25(ОН)D в крови рекомендуется оценивать через два-три месяца от начала лечения через три или более дней после последнего приема витамина D в дозе, превышающей 10 000 МЕ [60].

## ДОПОЛНИТЕЛЬНАЯ ИНФОРМАЦИЯ

Источник финансирования. Работа выполнена по инициативе авторов без привлечения финансирования.

Конфликт интересов. Все авторы одобрили финальную версию статьи перед публикацией, выразили согласие нести ответственность за все аспекты работы, подразумевающую надлежащее изучение и решение вопросов, связанных с точностью или добросовестностью любой части работы.
